# Supplementary material for: High-throughput sequencing to analyze changes in the human scalp microbiome during the use of a shampoo
Source: BMC Microbiol. 2025 Aug 11;25:497. doi: 10.1186/s12866-025-04260-5 (PMC12337528; doi:10.1186/s12866-025-04260-5)
Supplement: Supplementary file 1 — Supplementary Material 1 [file 12866_2025_4260_MOESM1_ESM.docx]

**Supporting information**

**High-throughput sequencing to analyze changes in the human scalp microbiome during the use of a shampoo**

Chong Xu^1^, Wenxi Li^1^, Lin Lin^1^, Dexiang Zhang^1^, Jinyu Lei^1^, Danyang Pan^1^, Shuangcheng Liang^2^, Yiti Chen^1^, Yuepeng Wan^1^, Jingyu He^1,3^*

**Table S1** Adonis test based on bray-curtis distances.

| Group | Bacterial | | Fungal | |
| --- | --- | --- | --- | --- |
|  | R^2^ | *P* | R^2^ | *P* |
| Day 0 vs Day 28 | 0.06896 | **0.015** | 0.04764 | **0.001** |

Bold values indicate statistically significant results, P < 0.05.

**Table S2** Relative abundance of top 10 dominant bacterial genus in different group

| Taxonomy | Staphylococcus | Cutibacterium | Pseudomonas | Lawsonella | Ralstonia | Pseudoalteromonas | Vibrio | Corynebacterium | Streptococcus | unidentified_Chloroplast |
| --- | --- | --- | --- | --- | --- | --- | --- | --- | --- | --- |
| Day0 | 16.55% | 11.58% | 6.20% | 10.08% | 5.56% | 10.57% | 9.47% | 1.38% | 3.56% | 1.87% |
| Day28 | 18.37% | 28.662% | 0.40% | 9.52% | 1.78% | 8.23% | 9.70% | 1.35% | 3.06% | 1.20% |

**Table S3** Relative abundance of top 10 dominant fungal genus in different group

| Taxonomy | Malassezia | Nigrospora | Aureobasidium | Pseudorhypophila | Aspergillus | Alternaria | Candida | Archaeorhizomyces | Fusarium | Wallemia |
| --- | --- | --- | --- | --- | --- | --- | --- | --- | --- | --- |
| Day0 | 81.34% | 1.80% | 0.01% | 2.28% | 0.69% | 0.49% | 0.08% | 1.19% | 1.01% | 0.15% |
| Day28 | 64.31% | 0.01% | 1.80% | 0.00% | 2.13% | 2.53% | 0.59% | 0.00% | 0.67% | 0.37% |

**Table S4** The table displays the relative pathway of bacterial prediction by PICRUSt2

| **Pathway** | **Description** | **Associated biosynthesis pathways** |
| --- | --- | --- |
| PHOSLIPSYN-PWY | superpathway of phospholipid biosynthesis I (bacteria) | Fatty acid and lipid biosynthesis |
| GLYCOLYSIS | glycolysis I (from glucose 6-phosphate) | Generation of precursor metabolites and energy |
| PWY-6125 | superpathway of guanosine nucleotides de novo biosynthesis II | Nucleoside and Nucleotide Biosynthesis |
| PWY-2942 | L-lysine biosynthesis III | Amino acid biosynthesis |
| PWY-7222 | guanosine deoxyribonucleotides de novo biosynthesis II | Nucleoside and nucleotide biosynthesis |
| PWY-7220 | adenosine deoxyribonucleotides de novo biosynthesis II |  |
| PWY-7229 | superpathway of adenosine nucleotides de novo biosynthesis I |  |
| TCA | TCA cycle I (prokaryotic) | Generation of precursor metabolites and energy |
| PWY-7219 | adenosine ribonucleotides de novo biosynthesis | Nucleoside and nucleotide biosynthesis |
| NONOXIPENT-PWY | pentose phosphate pathway (non-oxidative branch) | Generation of precursor metabolites and energy |
| PWY-3781 | aerobic respiration I (cytochrome c) |  |
| PWY-5484 | glycolysis II (from fructose 6-phosphate) |  |
| PWY-841 | superpathway of purine nucleotides de novo biosynthesis I | Nucleoside and Nucleotide Biosynthesis |
| PWY-7208 | superpathway of pyrimidine nucleobases salvage |  |
| PWY-7228 | superpathway of guanosine nucleotides de novo biosynthesis I |  |
| ANAGLYCOLYSIS-PWY | glycolysis III (from glucose) | Generation of precursor metabolites and energy |
| PWY4FS-8 | phosphatidylglycerol biosynthesis II (non-plastidic) | Fatty acid and lipid biosynthesis |
| PWY4FS-7 | phosphatidylglycerol biosynthesis I (plastidic) |  |
| PWY-6121 | 5-aminoimidazole ribonucleotide biosynthesis I | Nucleoside and nucleotide biosynthesis |
| PWY-6126 | superpathway of adenosine nucleotides de novo biosynthesis II |  |
| CALVIN-PWY | Calvin-Benson-Bassham cycle | Carbon consumption |
| THRESYN-PWY | superpathway of L-threonine biosynthesis | Amino acid biosynthesis |
| PWY0-1319 | CDP-diacylglycerol biosynthesis II | Phospholipid biosynthesis |
| PWY-5667 | CDP-diacylglycerol biosynthesis I |  |
| COMPLETE-ARO-PWY | superpathway of aromatic amino acid biosynthesis | Amino acid biosynthesis |
| PWY-3001 | superpathway of L-isoleucine biosynthesis I |  |
| FAO-PWY | fatty acid &beta-oxidation I | Generation of precursor metabolites and energy |
| PWY-7111 | pyruvate fermentation to isobutanol (engineered) |  |
| PWY-7663 | gondoate biosynthesis (anaerobic) | Fatty acid biosynthesis |
| BRANCHED-CHAIN-AA-SYN-PWY | superpathway of branched amino acid biosynthesis | Amino acid biosynthesis |
| FASYN-ELONG-PWY | fatty acid elongation -- saturated | Fatty acid biosynthesis |
| PWY-5101 | L-isoleucine biosynthesis II | Amino acid biosynthesis |
| PWY-5973 | cis-vaccenate biosynthesis | Fatty acid biosynthesis |
| VALSYN-PWY | L-valine biosynthesis | Amino acid biosynthesis |
| ILEUSYN-PWY | L-isoleucine biosynthesis I (from threonine) |  |

**Table S5** The table displays the trophic mode of fungal prediction by FUNGuild

| Trophic Mode | Day 0 | Day 28 |
| --- | --- | --- |
| Unassigned | 0.082195 | 0.248605 |
| Pathotroph-Saprotroph-Symbiotroph | 0.007919 | 0.043755 |
| Symbiotroph | 0.002846 | 0.000314 |
| Pathotroph-Symbiotroph | 0.001676 | 0.000536 |
| Pathogen-Saprotroph-Symbiotroph | 0.000248 | 5.60E-05 |
| Saprotroph | 0.069782 | 0.051706 |
| Saprotroph-Symbiotroph | 0.001636 | 0.000908 |
| Pathotroph-Saprotroph | 0.827825 | 0.650346 |
| Pathotroph | 0.005872 | 0.003773 |
