# Supplementary material for: High-throughput sequencing to analyze changes in the human scalp microbiome during the use of a shampoo
Source: BMC Microbiol. 2025 Aug 11;25:497. doi: 10.1186/s12866-025-04260-5 (PMC12337528; doi:10.1186/s12866-025-04260-5)
Supplement: Supplementary file 3 — Supplementary Material 3 [file 12866_2025_4260_MOESM3_ESM.pdf]

High-throughput sequencing to analyze changes in the human scalp microbiome during the use of a shampoo

Chong Xu[1,2], Wenxi Li[1], Lin Lin[1], Dexiang Zhang[1], Jinyu Lei[1], Danyang Pan[1], Shuangcheng Liang[3], Yiti Chen[1], Yuepeng Wan[1], Jingyu He[1,4]*

1 Research Center of New Material, Guangzhou Uniasia Cosmetics Technology Co., Ltd., Guangzhou 510663, China;

2 Guangdong Jiangmen Chinese Medicine College, Jiangmen 529000, China;

3 Research Center of Chinese Herbal Resource Science and Engineering, Guangzhou University of Chinese Medicine; Key Laboratory of Chinese Medicinal Resource from *Lingnan* (Guangzhou University of Chinese Medicine), Ministry of Education; Joint Laboratory of National Engineering Research Center for the Pharmaceutics of Traditional Chinese Medicines, Guangzhou, 510006, P. R. China;

4 School of Chemistry, South China Normal University, Guangzhou 510006, China

*Corresponding author. E-mail address: algerho1983@hotmail.com

Abstract

Shampoo, a crucial personal hygiene product, holds significant economic importance. Given the complex interactions of the scalp microbiome and its impact on scalp and hair health, understanding how shampoo affects this ecosystem is vital. Results showed that 28-day shampoo use significantly increased scalp moisture content（P = 0.04） without affecting sebum secretion（P = 0.23）. Regarding the bacterial community, while alpha diversity indices showed no significant changes, the relative abundance of *Proteobacteria* was decreased significantly, and the relative abundance of *Actinobacteriota* was increased significantly; the relative abundance of *Cutibacterium* genus was increased significantly, from 11.58% to 28.66%, more abundant than *Staphylococcus* genus (from 16.55% to 18.37%). For the fungal community, the Chao 1 index decreased significantly, and the relative abundances of several phyla and genera changed, but retained the core microbiome (*Malassezia*). Canonical correspondence analysis revealed that the microbial communities shifted from an oily environment to a more moisturized environment. Network analysis showed different co-occurrence relationships between core taxa and physicochemical factors for bacteria and fungi. Functional prediction via PICRUSt2 and FUNGuild indicated that the shampoo altered metabolic pathways of bacteria and the trophic modes of fungi. In conclusion, the study described the complex community dynamics of scalp microorganisms before and after 28-day shampoo use. The results will help improve the interactions among shampoo, scalp microbiota, and scalp physiology, offering valuable insights for the development of scalp and hair care products and strategies.

Keyword： High-throughput sequencing, microbial community, human scalp, shampoo, scalp health care

Clinical trial number

Not applicable.

Introduction

For most people, shampooing is nearly a daily routine. This is a seemingly mundane act that serves as a cornerstone of personal hygiene and grooming, which explains consumers' strong interest in shampoos. Indeed, shampoo occupies a dominant share of the hair wash and hair care markets [1, 2], reflecting its pervasive role in daily life. This widespread use underscores the critical need to understand how such frequent product application impacts the scalp microbiome.

Shampoo, in basic terms, is a product for cleaning the scalp and hair, and is usually consist of fundamental components such as surfactants, preservatives, conditioners, and active ingredients [3]. Many factors, including age, sex, genetics, environmental influences, and hair care products, etc., can influence the diversity and activity of the scalp's microbial community, which in turn is related to the scalp and hair health [4-6]. Among these, hair care products are an artificially and effectively extrinsic device. Therefore, the choice of an appropriate shampoo variety becomes particularly critical, as it directly affects the scalp microbiome and, ultimately, the health of the hair and scalp. Currently, there is a complete category of shampoo products on the market designed to meet the needs of consumers, including those for oil control, moisturizing, dandruff removal, and so on.

The impact of shampoo on scalp microecology has been taken seriously. The complex interactions among the human scalp and other associated organisms, including bacteria, fungi, viruses, and mites, are studied [7, 8]. These organisms can interact with each other in either a synergistic or antagonistic manner. On the one hand, the human scalp is home to a wide variety of microorganisms that play roles in health and defense, interacting as a single functional unit known as a holobiont. *Cutibacterium* and *Staphylococcus* are resident bacteria on the healthy human scalp that exist in a balanced state. *Cutibacterium* can inhibit *staphylococcal* growth through the secretion of antimicrobial peptides, while *staphylococcus* ferment glycerol to curb excessive *Cutibacterium* proliferation [9]. On the other hand, microorganisms may have negative effects on the human scalp, including disrupting scalp barrier function,

exacerbating the inflammatory response, and interfering with metabolic processes and nutrient cycling. *Cutibacterium* by its lipase activity can hydrolyze human sebum triglycerides and generate shorter-chain fatty acids that can maintain the weakly acidic environment on the surface of the scalp [10]. However, excessive reproduction and local accumulation of short-chain fatty acids can cause inflammation, which may affect healthy scalp [11].

To understand the importance of maintaining a balanced scalp microecology for scalp and hair health, it is essential to gain insight into the interactions between shampoo and microorganisms. For studying bacteria, PCR is frequently employed to amplify the widely present 16S ribosomal RNA (rRNA) gene. By sequencing its variable regions, precise taxonomic identification can be achieved. For studying fungi, the ribosomal internal transcribed spacer (ITS) is often used as the ideal marker sequence to profile the fungal community [12, 13]. Therefore, to explore the influence of shampoo on scalp microecology, the bacterial and fungal community structures and diversities before and after 28-day shampoo use were analyzed via 16S rDNA and ITS high-throughput sequencing in the present study. The findings in the investigations may shed light on the potential to inform the development of more targeted and microbiome-friendly scalp care products, ultimately improving scalp health and well-being.

Methods

Shampoo formula

The formula for a microbiome-balancing shampoo contained (% w/w): the fermentation filtrate of soapberry pericarp 0.5, ethanolamine pyrrolidone 0.1, cocamidopropyl betaine 10, decyl glucoside 2, sodium laureth sulfate 12, polyquaternium-10 0.6, PPG-3 octyl ether 0.5, cocamide methyl MEA 3, sodium benzoate 0.3, phenoxyethanol 0.3, sodium citrate 0.2, citric acid 0.2, disodium EDTA 0.1, fragrance 0.5, and water 69.7.

Volunteer recruitment

Twenty healthy volunteers (18 females and 2 males) were recruited from the human

efficacy laboratory at Guangzhou Uniasia Cosmetics Technology Co., Ltd. Participants were selected based on predefined inclusion and exclusion criteria. Inclusion criteria required volunteers to be aged 18–60 years, provide informed consent, and maintain consistent lifestyle habits throughout the study without using additional comparable products. Exclusion criteria included recent use of antihistamines or immunosuppressive agents, participation in other clinical trials within the past three months, allergies to study product components, pregnancy or lactation, recent cosmetic treatments, and a history of systemic, autoimmune, or immunodeficiency disorders. All procedures adhered to regulatory guidelines, and informed consent was obtained from all participants.

Treatment

The volunteers were asked to stop using any hair- or scalp-related products for at least three days before the treatment. During the trial, an appropriate amount of shampoo was applied to the palm, lathered, and applied to pre-wet hair. The shampoo was massaged thoroughly into the scalp and hair, left on for ten minutes, and then rinsed off. This procedure was performed three times a week for four weeks. The study was approved by Institutional Review Board of the Human Efficacy Laboratory at Guangzhou Uniasia Cosmetics Technology Co., Ltd. (No.UAISH230802003, approved in August 2023).

Scalp oil and moisture content assessments

The scalp oil and moisture content were measured using a scalp oil tester (Meibometer MB560, China) and a digital moisture tester (DermaLab Combo, Denmark), respectively. All measurements were conducted at a temperature of 25 ± 1°C and a relative humidity of 40 ± 5%. Group differences were analyzed via t-tests using R version 3.5.3.

Questionnaire of shampoo use

All volunteers completed a self-administered questionnaire on shampoo use aimed at evaluating the status of the human scalp, including the effect in reducing scalp irritation, antipruritic effect, overall soothing effect, effect in reducing scalp oiliness,

overall oil-control effect, effect of hydrating the scalp, and any signs of irritation or allergic response. All study participants had previously used the shampoo for 28 days. Each characteristic was rated on a 5-point Likert scale, where “strongly agree” was given 5 points, and “strongly disagree” was given 1 point.

Sample collection, DNA extraction and PCR amplification

Scalp samples were collected using sterile cotton swabs moistened with saline, applied to the designated area of the scalp for 30 seconds. The swabs were immediately placed in collection tubes, flash-frozen in liquid nitrogen, stored at −80°C, and transported to Novogene (Beijing, China) on dry ice.

Genomic DNA was extracted using the CTAB (cetyltrimethylammonium bromide) method, and DNA quality and concentration were assessed via 1% agarose gel electrophoresis and NanoDrop spectrophotometry, respectively. DNA was normalized to 1 ng/μL with sterile water. The V4 region of bacterial 16S rRNA and the ITS1 region of fungal rDNA were amplified using barcoded primers (16S: 515F/806R; ITS1: ITS1-5F/ITS1R). PCR reactions included 2 μM primers, 15 μL Phusion® High-Fidelity Master Mix, and ~10 ng template DNA, with thermocycling conditions: 98°C for 1 min, followed by 30 cycles of 98°C for 10 s, 50°C for 30 s, and 72°C for 30 s, with a final extension at 72°C for 5 min.

Library preparation and sequencing

Libraries were constructed using the TruSeq® DNA PCR-Free Sample Preparation Kit (Illumina, USA). Quantification was performed using Qubit fluorometry and quantitative PCR (qPCR), followed by sequencing on the NovaSeq PE250 platform (Illumina).

Bioinformatics and data analysis

Raw sequencing data underwent preprocessing to remove barcodes and primers. Paired-end reads (R1 and R2) were merged using Flash (Version 1.2.11) [14], and high-quality clean tags were generated via Fastp (Version 0.23.1) [15]. Chimera sequences were removed using vsearch (Version 2.16.0) [16]. Amplicon sequence variants (ASV) (with 100% clustering threshold) were obtained using DADA2 in

QIIME2 (version 202202) [17], and taxonomic annotations were performed against the Silva (Version 138.1, http://www.arb-silva.de/) [18] (bacterial 16S rRNA) and Unite (Version 9.0, https://unite.ut.ee/) [19] (fungal ITS) databases.

Alpha diversity indices (Chao 1, Shannon) and beta diversity indices (Bray-Curtis dissimilarity) were calculated using QIIME (version 1.9.1). Group differences in alpha diversity were analyzed via Kruskal-Wallis H tests, while beta diversity differences were assessed using non-metric multidimensional scaling (NMDS) on Bray-Curtis matrices. The linear discriminant analysis (LDA) was employed to evaluate significant taxonomic differences in bacteria and fungi. The method was the same as previously described [20]. The relationships between the microbial community and physicochemical factors were analyzed using canonical correspondence analysis (CCA) in R (version 4.3.1). Co-occurrence network analysis was conducted using the R igraph package, and Gephi software was used for visualization. All statistical significance was set at $P< 0.05$.

Results

Effect of a shampoo on oil values and moisture content in the human scalp

To investigate the effect of the shampoo on the human scalp, oil values and moisture content were determined on Day 0 and Day 28 (Fig. 1A). The oil values were slightly higher on Day 0 (average=516.90) than on Day 28 (average=474.60), showing an 8.18% decrease that was not significant difference ($P > 0.05$). In contrast, the moisture content was considerably lower on Day 0 (average=38.02) than on Day 28 (average=50.32), showing statistically significant with a 32.35% increase that was ($P < 0.05$) (Fig. 1B). Overall, the 28-day use of the shampoo had a notable impact on scalp moisture content but did not significantly affect scalp oil production. Interestingly, volunteers perceived that the effect of reducing scale oiliness was greater than the effect of hydrating the scale based on questionnaire surveys (Fig. 1C). These findings suggest that the shampoo may be beneficial for both improving scalp hydration and reducing scalp oiliness.

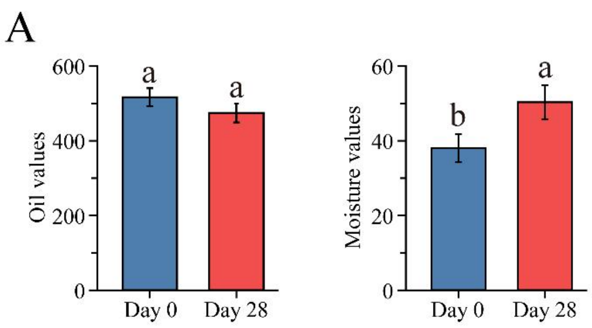

B

Group	Oil values			Moisture values		
	Mean	R^2	*P*	Mean	R^2	*P*
Day 0	516.90	0.04	0.23	38.02	0.10	**0.04**
Day 28	474.60			50.32		

Bold values indicate statistically significant results, $P < 0.05$.

C

Characteristic	Total	Min value	Max value	Mean	Stander error of mean
Effect in reducing scalp irritation	20	1	4	2.75	0.18
Antipruritic effect	20	1	4	2.55	0.18
Overall soothing effect	20	2	4	2.80	0.14
Effect in reducing scalp oiliness	20	1	4	2.95	0.18
Overall oil-control effect	20	2	4	3.05	0.15
Effect of hydrating the scalp	20	1	4	2.75	0.18
Irritation or an allergic response	20	0	0	0	0

Figure 1. Analysis of oil values and moisture content of the human scalp between the Day 0 and Day 28 groups. A, Changes in oil values and moisture content in the human scalp after using the shampoo. Bars represent the mean ± stander error of mean, n=20. B, T-test on oil values and moisture content for the Day 0 and Day 28 groups. C, The result of shampoo use based on questionnaires completed by volunteers.

Effect of a shampoo on the bacterial community of the human scalp

After 28-day use, the composition of the bacterial community on the human scalp showed significant variation. The results shown in Figure 2, the Chao 1 index, which reflects community richness, showed no significant difference between Day 0 and Day 28 ($P = 0.5261$). Similarly, the Shannon index, which measures diversity, also did not show a significant change ($P = 0.2332$). However, there was a slight decrease in the median values of both indices on Day 28 compared to that on Day 0, suggesting a

potential trend toward reduced diversity and richness, although this change was not statistically significant. Non-metric multidimensional scaling analysis (NMDS) based on Bray-Curtis distance showed significantly different clustering of the microbial community in the Day 0 and Day 28 groups (Figure 2B). This result was further confirmed by the permutational multivariate analysis of variance (Adonis test), which was also calculated using the Bray-Curtis distance matrix (Supplementary Table S1). The relative abundances of bacterial phyla and genera were performed to understand the dynamic changes on the bacterial community of the human scalp. At the phylum level, *Firmicutes*, *Proteobacteria*, *Actinobacteriota*, *Bacteroidota*, and *Cyanobacteria* dominated the human scalp in both the Day 0 and Day 28 groups. Among them, the relative abundances of *Proteobacteria* decreased significantly, whereas *Actinobacteriota* increased significantly from Day 0 to Day 28 (Figure 2C). At the genus level, *Staphylococcus* was the predominant genus in the Day 0 group, succeeded by *Cutibacterium*, and *Pseudoalteromonas* (Figure 2D and Supplementary Table S2). However, after 28-day use, a notable shift took place. The proportion of *Cutibacterium* increased significantly, resulting in *Cutibacterium* becoming more abundant than *Staphylococcus* in the Day 28 group. Moreover, the relative abundances of *Pseudomonas* and *Ralstonia* were reduced in comparison to those in the Day 0 group (Supplementary Table S2). To delve deeper into the differences observed on Day 0 and Day 28, linear discriminant analysis (LDA) was employed. The LDA identified 9 bacterial taxa with significant differences. Four bacterial taxa were significantly enriched on Day 0, mainly genera such as *Acinetobacte*r, while five bacterial taxa were significantly enriched on Day 28, including genera like *Stenotrophomonas*, *Sphingomonas*, and *Enhydrobacter* (Figure 2E). These findings reveal the potential advantages associated with the use of this shampoo, indicating its influence on the composition of the scalp bacterial microbiome.

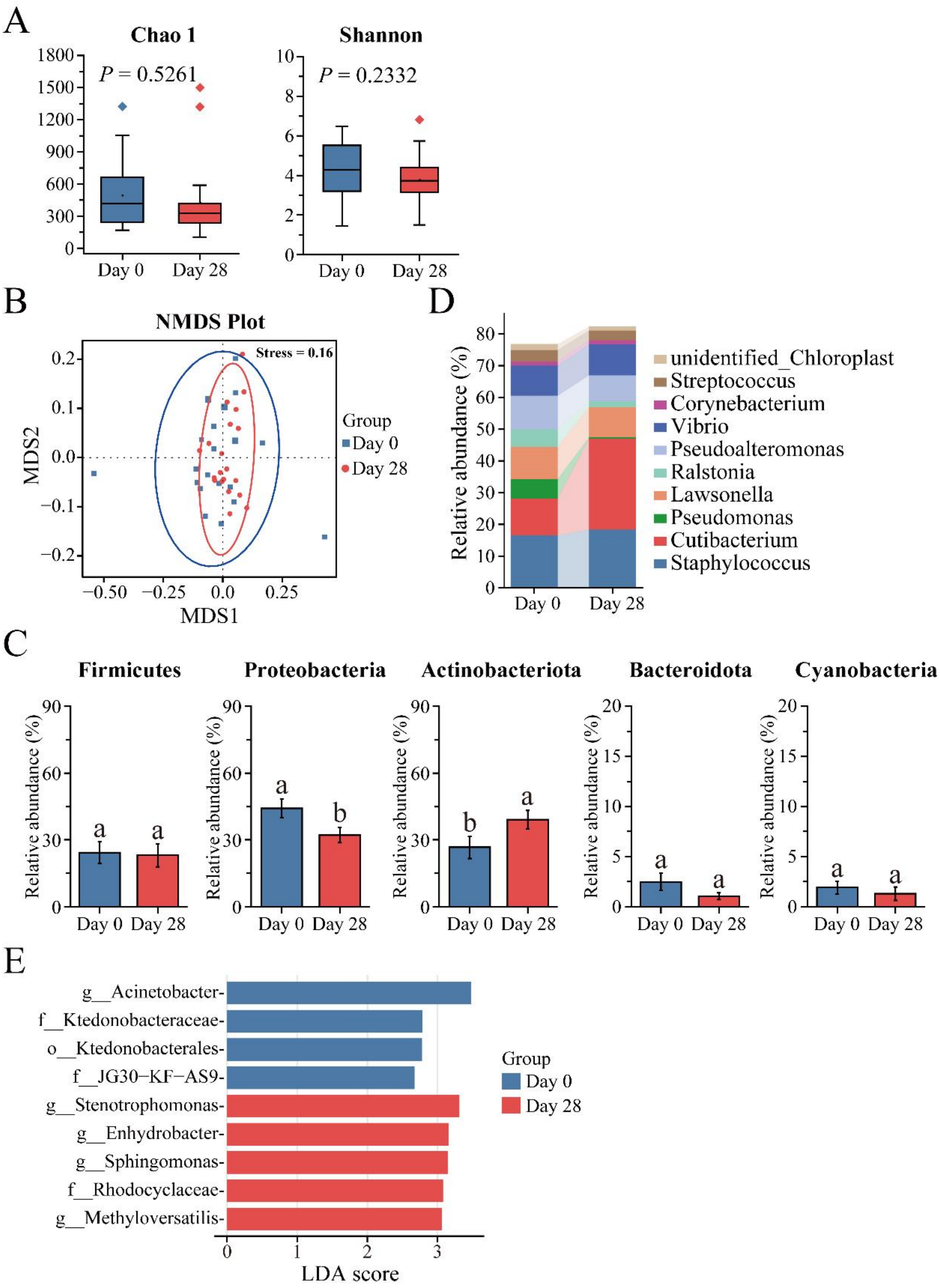

Figure 2. Effect of a shampoo on the bacterial community of the human scalp. A, Chao 1 index and Shannon index (alpha diversity). The boxplots display the minimum, lower quartile (Q1), median, upper quartile (Q3), and maximum values. The upper whisker represents the maximum value, the top line of the box indicates Q3 (75%), the central line of the box represents the median, the bottom line of the box indicates

Q1 (25%), and the lower whisker indicates the minimum. Outliers are shown as points outside the box-and-whisker structure; B, NMDS analysis of bacterial structures across Day 0 to Day 28 (beta diversity). Stress value is less than 0.2 indicates that the NMDS can accurately reflect the differences between samples; C, Compositional differences in relative abundance at the phylum level in the Day 0 and Day 28 groups; D, Compositional differences in relative abundance at the genus level in the Day 0 and Day 28 groups. Bars represent the mean ± stander error of mean; E, The LDA scores of the microbial communities were significantly different between Day 0 and Day 28 groups. Blue and red indicate taxa enriched in Day 0 and Day 28 groups, respectively.

Effect of a shampoo on the fungal community of the human scalp

Beyond bacteria, fungi also play pivotal roles in maintaining the human scalp microenvironment. The alpha diversity analysis showed that the Chao 1 index decreased significantly from Day 0 to Day 28 ($P = 0.0005$), while the Shannon index did not display a statistically significant change ($P = 0.7291$) (Figure 3A). This finding indicated that despite the decline in community richness, the overall diversity of the fungal community within the scalp microenvironment remained relatively stable. NMDS based on Bray-Curtis distance generated distinct clusters between the Day 0 and Day 28 groups, indicating a significantly differences in the distribution of fungal communities after using shampoo for 28 days (Figure 3B). Furthermore, the relative abundances of fungal phyla and genera were also examined to understand the dynamic changes in the fungal community of the human scalp. At the phylum level, the human scalp is mainly composed of *Basidiomycota*, *Ascomycota*, *Mortierellomycota*, *Zoopagomycota*, and *Rozelomycota*. Compared with the Day 0 group, the relative abundances of *Basidiomycota*, *Mortierellomycota*, and *Rozelomycota* were significantly lower ($P <0.05$) in the Day 28 group. Additionally, *Ascomycota* and *Zoopagomycota* exhibited a decrease, with no statistically significance (Figure 3C). At the genus level, *Malassezia* was the predominant fungal

genus in both Day 0 (81.34%) and Day 28 (64.31%) groups. Other genera, such as *Nigrospora*, *Aureobasidium*, *Pseudorhaphophila*, and *Aspergillus* also presented different relative abundances between the Day 0 and Day 28 groups. The relative abundances of some genera, like *Nigrospora* and *Pseudorhaphophila* showed a decrease from Day 0 to Day 28, while those of *Aureobasidium* and *Aspergillus* increased (Figure 3D and Supplementary Table S3). To further study the differences between Day 0 and Day 28, linear discriminant analysis (LDA) was employed. The LDA identified 27 fungal taxa with significant differences. Fifteen fungal taxa were significantly enriched on Day 0, mainly in *Pseudorhypophila* and *Archaeorhizomyces*, while twelve fungal taxa were significantly enriched on Day 28, primarily in *Neoascochyta* and *Epicoccum*. Overall, these results imply that the shampoo has the potential ability to adjust the composition of the scalp fungal microbiome.

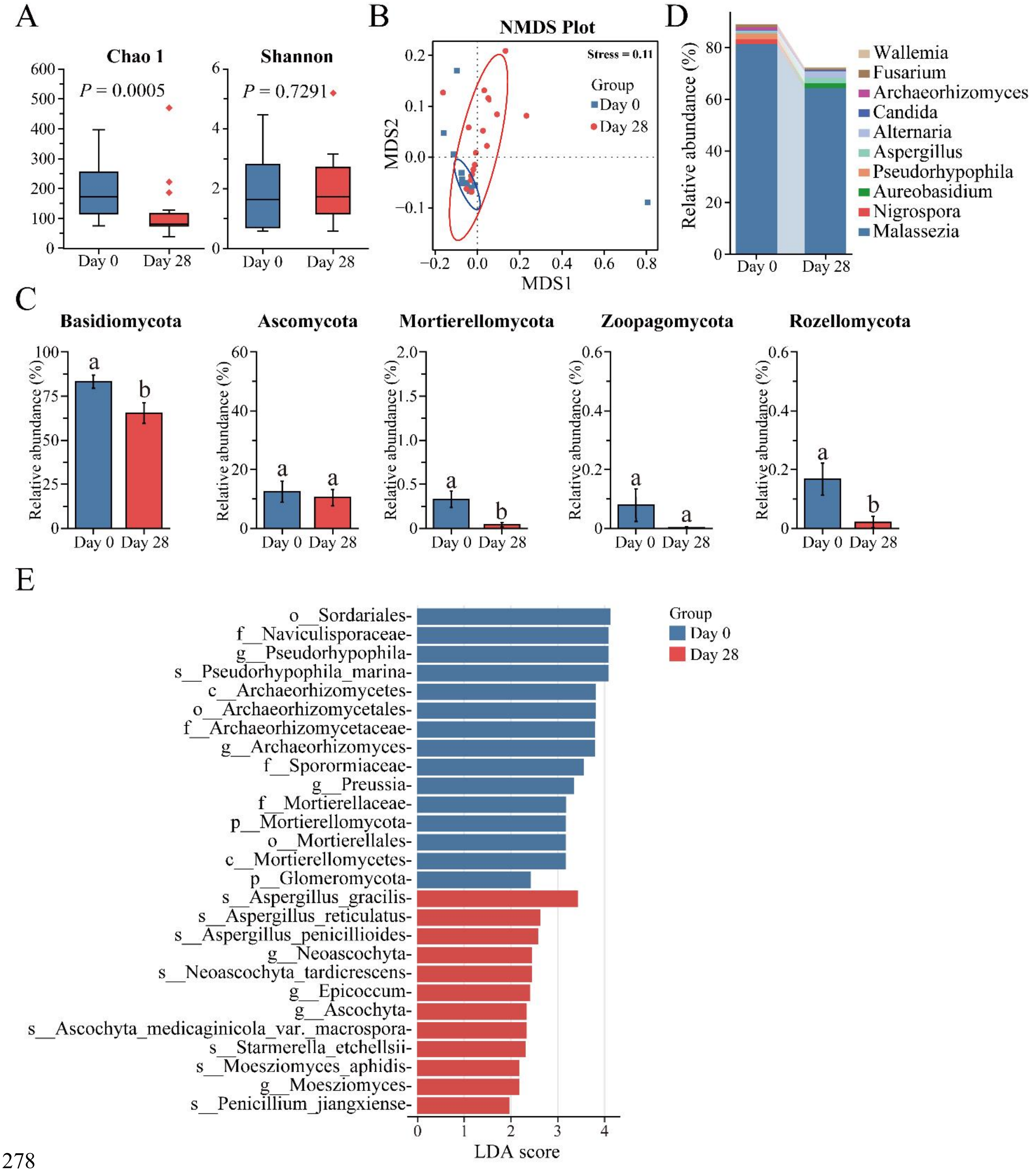

Figure 3. Effect of a shampoo on the fungal community of the human scalp. A, Chao 1 index and Shannon index (alpha diversity). The boxplots display the minimum, lower quartile (Q1), median, upper quartile (Q3), and maximum values. The upper whisker represents the maximum value, the top line of the box indicates Q3 (75%), the central line of the box represents the median, the bottom line of the box indicates Q1 (25%), and the lower whisker indicates the minimum. Outliers are shown as points

outside the box-and-whisker structure; B, NMDS analysis of fungal structures across Day 0 to Day 28 (beta diversity); C, Compositional differences in relative abundance at the phylum level in the Day 0 and Day 28 groups; D, Compositional differences in relative abundance at the genus level in the Day 0 and Day 28 groups. Bars represent the mean ± stander error of mean. E, The LDA scores of the microbial communities were significantly different between Day 0 and Day 28 groups. Blue and red indicate taxa enriched in Day 0 and Day 28 groups, respectively.

Correlation analysis of the microbial community with physicochemical factors

The microbial community of the human scalp was closely related to the physicochemical factors on the scalp. After 28-day shampoo use, the oil value was decreased, while the moisture content was increased with statistical significance. Canonical correspondence analysis (CCA) was employed to explore the influence of the shampoo on the variation of the human scalp microbial communities (Figure 4). The result showed that the first axis (CCA1) explained 60.27% of the variation and has positive correlation with moisture and negative correlation with oil; whereas the second axis (CCA2) explained 39.73% of the variation and positive correlation with oil and moisture. Samples collected on Day 0 were predominantly concentrated on the left side, which is relatively closer to the starting point of the oil arrow. In comparison, samples obtained on Day 28 are chiefly concentrated on the right side, which is relatively nearer to the starting point of the moisture arrow (Figure 4A). This implied that the bacterial communities changed after 28-day shampoo use, with a notable shift from an oily scalp environment to one with higher moisture. In figure 4B, the result reveals that the first axis (CCA1) explained 52% of the variation, negatively correlating with oil and moisture. The second axis (CCA2) accounted for 48% of the variation, positively correlating with moisture and negatively correlating with oil. Samples collected on Day 0 were predominantly concentrated on the bottom, closer to the oil arrow, and samples obtained on Day 28 were mainly at the up, nearer to the

moisture arrow (Figure 4B). These results are similar to the bacterial community, demonstrating a significant shift from an oily to a more moisture scalp environment.

Network analysis was conducted to investigate the co-occurrence relationships among the core taxa and physicochemical factors, relying on the strong correlation coefficients. A more densely connected module was generally observed in the bacteria (average path length = 2.674) than in fungi (average path length = 2.851), as shown in Figure 4C and 4D. Among them, some microbial taxa were significantly associated with oil or moisture physicochemical factors. For bacteria, *Varibaculum*, *Pseudomonas* and *Rubrobacteriaceae* were positively correlated with oil, while *Comamonas*, *Propionimicrobium*, and *Methanobacterium* were positively correlated with moisture. Notably, an unidentified genus and *Exiguobacterium* were negatively correlated with both oil and moisture. For fungi, *Malassezia* and *Scleroderma* were positively correlated with oil, and *Aspergillus* and *Starmerella* were positively correlated with moisture. Interestingly, two unidentified genera also exhibited negative correlations with both oil and moisture (Additional file 1).

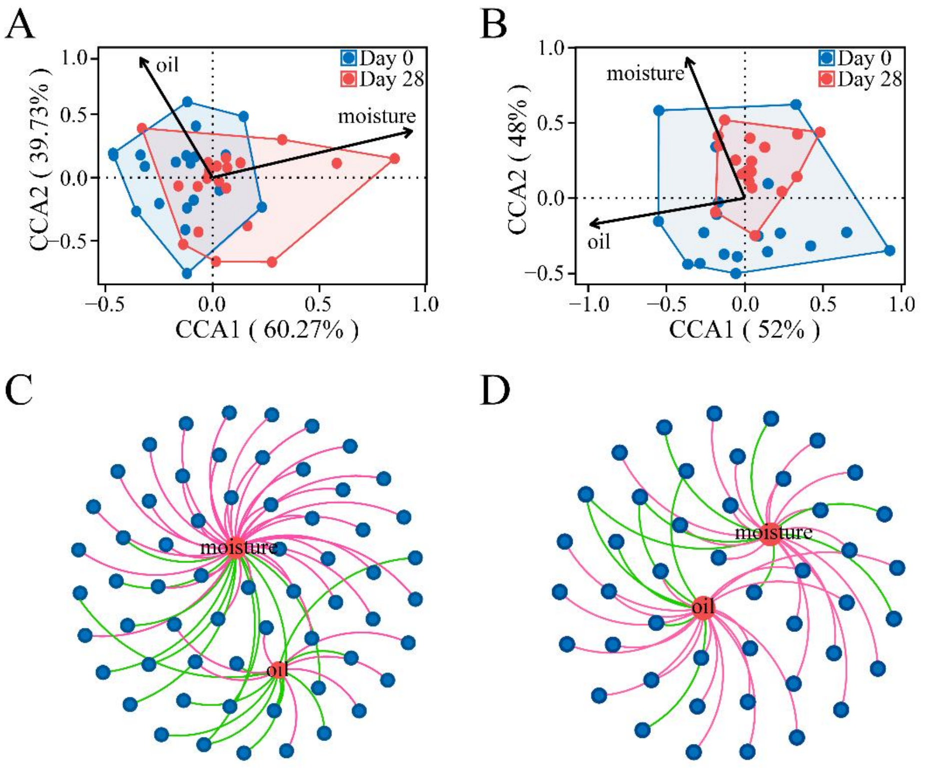

Figure 4. Correlation analysis between microbial community and physicochemical factors. A and B represent the relationship between bacteria and fungal communities

in CCA with physicochemical factors, respectively; C and D represent the co-occurrence network of physicochemical factors with bacteria and fungi, respectively. Blue ellipse nodes represent microbial members. Red ellipse nodes represent physicochemical factors. The connections between nodes indicate strong correlations (Spearman correlation coefficient, $|r| \geq 0.6$; $P < 0.001$). The color of the edges represent positive correlation (red) or negative correlation (green).

Functional prediction of the fungal and bacterial taxa through PICRUSt2 and FUNGuild

To better understand the effects of the shampoo after 28-day use, we predicted the function of the bacterial and fungal microbiota involved in the groups. Through the PICRUSt2 analysis (Figure 5A), a total of 442 unique MetaCyc pathways were predicted, and the 35 most abundant pathways were displayed using a heat map. Among them, the predicted functional analysis in all groups primarily found associations with amino acids, nucleosides, nucleotides, fatty acids, and the generation of precursor metabolites and energy. Eleven pathways were significantly enriched on Day 0, including gondoate biosynthesis (anaerobic), pyruvate fermentation to isobutanol (engineered), fatty acid chain elongation (saturated), etc. In contrast, the remaining twenty-four pathways, which are the glycolysis, the TCA cycle, phosphatidylglycerol biosynthesis, adenosine deoxyribonucleotide de novo biosynthesis II, guanosine deoxyribonucleotide de novo biosynthesis II, and others were enriched on Day 28 (Figure 5A and Supplementary Table S4). However, it is important to note that PICRUSt2-based functional predictions have inherent limitations compared to metagenomic and metatranscriptomic analyses, as they cannot directly capture real-time microbial gene expression or functional activity. This implies that the metabolic pathways changed after 28-day shampoo use, suggesting potential variations in metabolic functions between Day 0 and Day 28 groups.

According to the FUNGuild analysis (Figure 5B), nine trophic modes were detected on both Day 0 and Day 28 groups, with pathotroph-saprotroph being the most abundant, followed by unassigned and saprotroph. The relative abundance of fungal

functions varied significantly after 28-day shampoo use. Compared with Day 0, the unassigned trophic modes and pathotroph-saprotroph-symbiotroph were increased on Day 28; however, the other trophic modes, including pathotroph-saprotroph, saprotroph, pathotroph, symbiotroph, pathotroph-symbiotroph, saprotroph-symbiotroph, and pathogen-saprotroph-symbiotroph, decreased on Day 28 (Figure 5B and Supplementary Table S5). Notably, FUNGuild trophic mode assignments rely on annotated fungal genes and may have limitations in resolving complex symbiotic relationships or uncharacterized species. As a result, we had to accept some unassigned trophic modes in the data. Overall, these findings indicate that the shampoo significantly influences the functional characteristics of the fungal community on the human scalp.

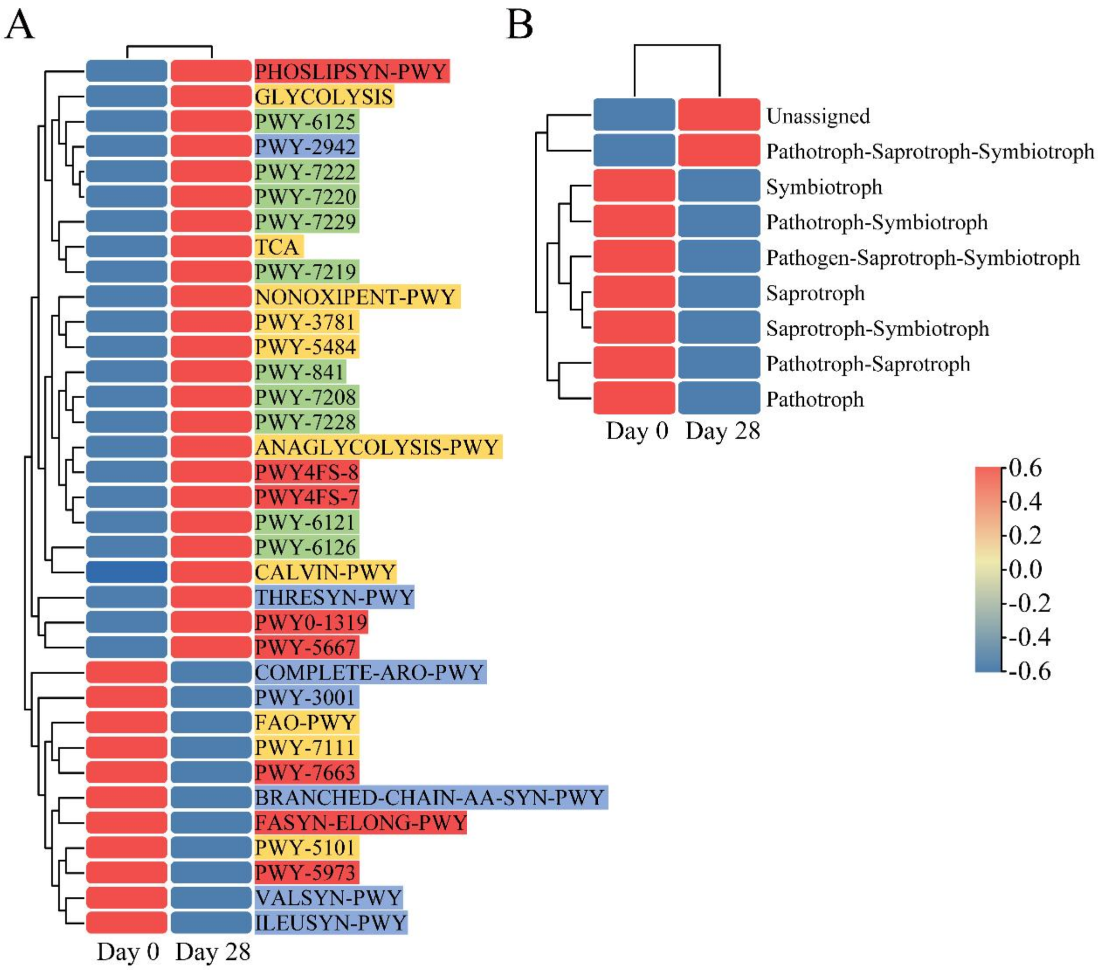

Figure 5 Functional prediction analysis of bacterial and fungal communities after 0 and 28 days of shampoo use (PICRUSt2 and FUNGuild). A, PICRUSt2 function prediction analysis for the bacterial community. Characters filled in red represents

fatty acid and lipid biosynthesis; Characters filled in yellow represents the generation of precursor metabolites and energy or carbon consumption; Characters filled in green represents nucleoside and nucleotide biosynthesis; Characters filled in blue represents amino acid biosynthesis. B, FUNGuild function prediction analysis for the fungi community.

Discussion

This study investigated the effects of using shampoo for 28 days on scalp oil and moisture content, as well as on the scalp microbiome. Our findings revealed changes in the levels of oil and moisture, along with alterations in the bacterial and fungal communities. These results provide valuable insights into the potential impacts of the shampoo on scalp condition and the microbiome.

Effects of the shampoo on human scalp condition

Initially, the primary purpose of shampoo was to remove soil to produce a clean scalp. However, the functions of shampoo have become more and more diverse, as the people's demands for beauty have evolved. Beyond its basic cleansing function, modern shampoos now offer a wide range of additional benefits, including moisturizing, oil control, dandruff removal, and hair repair, among others. In the current study, we found that this shampoo could ameliorate scalp conditions in humans. Improvements, including slightly decreased oil secretion and significantly increased moisture content, were observed after 28-day shampoo use (Figure 1A). The results indicate that the shampoo could enhance scalp hydration while maintaining the natural oil balance. This aligns with previous research that reported the certain moisturizing shampoos could boosting scalp moisture levels without disturbing the natural sebum balance [21]. Such a balance is crucial for maintaining overall scalp health, as excessive oiliness can lead to issues such as dandruff and scalp irritation, and insufficient moisture can result in dryness and flakiness [22]. Thus, the shampoo in our study provides further evidence of the potential for modern shampoo formulations to achieve a harmonious balance between hydration and oil regulation. Nevertheless, it is essential to recognize the limitations of sample size and study

duration in this study. Future research with larger populations and longer-term follow-up periods would be necessary to confirm the creditable efficacy of the shampoo as daily product.

Effects of the shampoo on microbial community structure

Previous studies have shown that coconut oil has a positive impact on scalp microbial communities and their function, and can affect scalp physiology, such as decreasing trans-epidermal water loss (TEWL) and increasing hydration, providing benefits to the human scalp [23]. In the current study, the shampoo could modify the scalp condition; thus, it seems reasonable to speculate that the shampoo has a potent effect on the scalp microbiome. Based on the major microbial species in the human scalp, the microbial community has been divided into bacteria and fungi. In the bacterial community, both the Chao 1 and Shannon indices show a slight decrease on Day 28 compared to Day 0, but this change was not statistically significant (Figure 2A). This might imply that the shampoo does not cause drastic shifts in the scalp microbial community; rather, there is a potential trend towards reducing richness and diversity, which is consistent with the NMDS analysis. The Day 0 and Day 28 samples were clustered together, and the circled region was reduced on Day 28 compared to Day 0. Metagenomic analysis showed that *Actinobacteria*, *Firmicutes* and *Proteobacteria* were the dominant phyla, while Cutibacterium and Staphylococcus were the dominant genera found on human scalp [9, 20, 24, 25]. This is the same as the results of this study. Notably, this study observed that the relative abundance of *Proteobacteria* decreased significantly, while that of *Actinobacteriota* increased significantly (Figure 2C). A possible explanation for this observation is that *Proteobacteria*, which include many opportunistic pathogens, like *Pseudomonas*, *Lawsonella* and *Ralstonia* genera, all of them thrive under certain imbalanced scalp conditions [26]. However, the shampoo may inhibit harmful microorganisms, thereby reducing the levels of *Proteobacteria*. In contrast, *Actinobacteria* are known for their metabolic versatility, capable of degrading complex organic compounds present on the scalp, including sebum and dead skin cells, which facilitate their growth and reproduction [27, 28].

This suggests that shampoo may create an environment conducive to the thriving of *Actinobacteria* microbiota.

In the fungi community, the Chao 1 index shows a dramatic decrease, while the Shannon index shows a slight increase from Day 0 to Day 28 (Figure 3A). This result corresponds to NMDS analysis, in which Day 0 and Day 28 samples were relatively scattered, and the circled region enlarged on Day 28 compared to Day 0 (Figure 3B). This implies that the shampoo could reduce community richness but stabilize overall fungal diversity. This could mean that the number of species were lost, the dominant species maintained their relative dominance, thus recognizing the proportionally larger role of dominant species in affecting ecosystem properties in relation to subordinate species [29, 30]. At present, many studies have shown that *Basidiomycota* and *Malassezia* as the main phylum and core genera found on healthy scalps [31], which is consistent with the findings obtained in this study. Interestingly, it was found that the *Nigrospora* and *Pseudorhaphophila* genera were decreased, and the *Aureobasidium*, *Aspergillus*, and *Alternaria* genera were increased after 28-day shampoo use. *Nigrospora* displays a certain pathogenic capability in many different ecological niches. In plants, the fungi of the *Nigrospora* genus cause squirter disease on bananas and stem blight disease on a variety of hosts, including rice, brown mustard, and tea [32-36]. In humans, the fungi of the *Nigrospora* genus cause human infections, referring to respiratory allergic reactions as well as skin, nail, and eye infections [37, 38]. Based on the commonalities of pathogenic microorganisms, it can be speculated that the fungi of the *Nigrospora* genus may trigger inflammatory reactions and have a negative impact on scalp health, when the balance of scalp microecology is disrupted. This suggests that the shampoo could reduce the potentially harmful fungi, helping to prevent or alleviate scalp infections and allergic reactions. There may be a symbiotic relationship between the fungi of the *Pseudorhaphophila* and *Nigrospora* genus, as the trend in the numbers of *Pseudorhaphophila* and *Nigrospora* genera is decreased. Additionally, from Day 0 to Day 28, the *Aureobasidium*, *Aspergillus*, and *Alternaria* genera increased from 0.01%, to 1.80%, 0.69% to 2.13%, and 0.49% to 2.53%, respectively (Supplementary table

S3). These fungi are commonly found on the skin and scalp. As long as they don't overgrow, there won't lead to health issues. Consequently, the shampoo exerts a positive effect on the scalp by modulating the scalp fungal community. However, individual differences in scalp microecology should be considered, as the response to the shampoo may vary among different individuals, living and working environments.

Physicochemical factors of the scalp in relation to microbial community structure and microbial interactions

The scalp moisture content was significantly increased and oil values was slightly decreased, with the dynamic changes in the composition of bacterial and fungal communities after using the shampoo for 28 days (Figure 1). These findings strongly suggest that the physicochemical factors play a pivotal role in shape the scalp microbiome. Previous studies have established that the ideal skin condition is characterized by a balanced water-oil ratio and better skin barrier function, that is, higher skin hydration content and lower sebum levels [39]. In the present study, the bacterial genera *Corynebacterium*, *Cutibacterium,* and *Pseudoalteromonas* showed a positive correlation with scalp moisture. *Corynebacterium* and *Pseudoalteromonas* are commonly found in water environments [40-42]. Whereas *Cutibacterium*, a lipophilic species, predominantly inhabits the skin. Its high correlation with moisture might be attributed to the fact that moderate moisture levels enhance the accessibility of nutrients, thereby facilitating its metabolic activities and proliferation [43]. This indicates that the increased scalp moisture after 28-day shampoo use is moderate without causing excessive. Some fungi also showed a positive correlation with moisture such as *Alternaria* and *Unidentified_fungi.* Generally, numerous fungi prefer environmental conditions with high relative humidity [44], which supports the results showing a slight increase in the Shannon index, suggesting an enhancement in ecosystem stability (Figure 3A). In contrast, the bacteria *Staphylococcus*, *Pseudomonas*, and *Vibrio*, as well as the fungi *Malassezia* and *Pseudorhaphophila marina*, showed a preference for oil conditions. Notably, this study revealed that after 28-day shampoo use, the scalp condition significantly changes from an oily

environment to a more moisturized environment (Figure 4). This change coincided with the reduced oil-preferring microorganisms, aligning with the decreased Chao 1 index of both bacterial and fungal communities. Overall, shampoo application facilitated the transition of the scalp microenvironment from an oily to a more moisture state, resulting in substantial changes in the dominance and relative abundance of microbial groups. These results indicate that even subtle changes in scalp moisture and sebum levels can act as selective pressures, driving alterations in the microbial community composition and structure.

Components of the shampoo in relation to microbial community structure

Understanding the composition of shampoo ingredients and their impact on the human scalp microbial community is crucial, as these microbes can directly influence the scalp microbiome and overall health [45]. Shampoo ingredients affect the microbial composition during use. Hence, studying the effect of different ingredients will help us understand the relationship between shampoo and scalp microbes, and ultimately develop scalp and hair care products related to 'scalp microecology'. In this study, the effect on the human scalp microbial community structure before and after 28-day shampoo use was investigated (Figure 2 and Figure 3).

Generally, the basic components of shampoo are surfactants, preservatives, conditioners, and active ingredients. In this study, cocamidopropyl betaine, decyl glucoside, and sodium laureth sulfate are the surfactants in the shampoo, which clean by lowering water's surface tension to remove grease [46]. Cocamidopropyl betaine and decyl glucoside are mild and have minimal antimicrobial effects, while sodium laureth sulfate is a strong anionic surfactant with notable antimicrobial activity against bacteria and fungi, potentially causing microbial imbalance with long-term use [47]. The preservatives, sodium benzoate and phenoxyethanol, prevent microbial contamination and extend shelf life; sodium benzoate inhibits bacterial and fungal growth such as *Staphylococcus aureus*, *Candida albicans* and

Aspergillus niger [48, 49]; while phenoxyethanol offers broad-spectrum antimicrobial protection even at low concentrations [50]. The conditioners, polyquaternium-10 and PPG-3 octyl ether, enhance hair smoothness and reduce static without significantly affecting the microbial community [51]. The fermentation filtrate of soapberry pericarp contributed to the efficacy of the shampoo, can affected microbial diversity and composition, as demonstrated in our previous study [20]. At different stages of using the fermentation filtrate of soapberry pericarp, the human scalp hosts different microbes. From Day 0 to Day 7 and Day 28, *Actinobacteriota* and *Firmicutes* were increased, while *Proteobacteria* was decreased [20]. In this study, an increase in *Actinobacteriota* and a decrease in *Proteobacteria* were found from Day 0 to Day 28, together with no significant change in *Firmicutes*. The changing trends of *Actinobacteriota* and *Proteobacteria* are consistent with the previous study. Therefore, it was speculated that the trend of changes in the human scalp was the same at different stages of shampoo use, but there was transient microbiome instability. Unfortunately, the early post-shampoo phases, such as Day 7 and Day 14, were not studied. Different components influenced the different microorganisms. By complex formulation study, the efficacy of the shampoo, which minimized interference with the normal microbiota and complies with the 'microbiota balance' was evaluated.

Conclusion

High-throughput sequencing technology is used to systematically explore the impact of shampoo on the human scalp microecology. After 28-day shampoo use, the scalp condition significantly changes from an oily environment to a more moisturized environment. The scalp microbiota (bacterial and fungal) underwent notable compositional changes, such as a decrease in *Proteobacteria* and an increase in *Actinobacteriota*. Additionally, the functional characteristics of the scalp microbiota was changed, altering bacterial metabolic pathways and fungal trophic modes. Therefore, shampoo may influence the scalp condition, further affecting the composition and functions of scalp-associated microbial communities. These findings

provide a scientific basis for developing a new generation of scalp and hair care products focused on "scalp microecology". As the study with limited populations and duration, future research should include extensive longitudinal data and multi-omics approaches, such as metagenomics and metabolomics, which are warranted to determine the interactions between shampoo, scalp microbiota, and scalp physiology as an effective strategy for scalp and hair care products in humans.

Acknowledgments

We would like to thank Hui He for assistance in the field sampling.

Author contributions

CX completed statistical analysis, manuscript writing and modification of the manuscript. WL, LL, and DZ were responsible for recruiting volunteers. JL, DP, and YC were responsible for sample collection. SL participated in manuscript writing and modification of the manuscript. YW and JH designed the study. JH participated in the modification of the manuscript;

Funding

This work was supported by the Guangdong S&T Program Project (2024B1111160002).

Data availability statement

The datasets analysed during the current study are available in the NCBI repository. The bacterial Illumina sequence data and the fungal Illumina sequence data can be accessed from the NCBI SRA under BioProject number PRJNA1268597 and PRJNA1270203, respectively.

Declarations

Ethics approval and consent to participate

The study was conducted according to the guidelines of the Declaration of Helsinki and was approved by Institutional Review Board of the Human Efficacy Laboratory at Guangzhou Uniasia Cosmetics Technology Co., Ltd. (No.UAISH230802003, approved in August 2023). Participants provided written informed consent prior to

taking part in the study.

Consent for publication

Not applicable.

Competing interests

The authors declare no competing interests.

References

1. Consulting SR: **Report on the Market Supply and Demand Situation and Investment Prospects of China's Shampoo Industry from 2024 to 2030**. In.; 2023.
2. Intelligence Q: **The Guide to Scientific Hair Washing and Hair Care in 2024**. In. wuhan; 2024.
3. Kligman AM, Mcginley KJ, Leyden JJ: **Studies on the Effect of Shampoos on Scalp Lipids and Bacteria**. *Springer Berlin Heidelberg* 1981.
4. Watanabe K, Yamada A, Nishi Y, Tashiro Y, Sakai KJSR: **Host factors that shape the bacterial community structure on scalp hair shaft**. 2021, **11**.
5. Oh J, Conlan S, Polley EC, Segre JA, Kong HH: **Shifts in human skin and nares microbiota of healthy children and adults**. *Genome Med* 2012, **4**(10):77.
6. Dimitriu PA, Iker B, Malik K, Leung H, Mohn WW, Hillebrand GG: **New Insights into the Intrinsic and Extrinsic Factors That Shape the Human Skin Microbiome**. 2019, **10**(4):10.1128/mbio.00839-00819.
7. Oh J, Deming C, Kong HH, Byrd AL, Conlan S, Segre JAJN: **Biogeography and individuality shape function in the human skin metagenome**. 2014.
8. Chen YE, Fischbach MA, Belkaid Y: **Skin microbiota-host interactions**. *Nature* 2018, **553**(7689):427-436.
9. Xu Z, Wang Z, Yuan C, Liu X, Yang F, Wang T, Wang J, Manabe K, Qin O, Wang XJR: **Dandruff is associated with the conjoined interactions between host and microorganisms**. 2016, **6**:24877.
10. Nakase K, Momose M, Yukawa T, Nakaminami H: **Development of skin sebum medium and inhibition of lipase activity in Cutibacterium acnes by oleic acid**. *Access Microbiol* 2022, **4**(10):acmi000397.
11. Corvec S: **Clinical and Biological Features of Cutibacterium (Formerly Propionibacterium) avidum, an Underrecognized Microorganism**. *Clin Microbiol Rev* 2018, **31**(3).
12. Turner TR, James EK, Poole PS: **The plant microbiome**. *Genome Biol* 2013, **14**(6):209.
13. Liang Z, Liu F, Wang W, Zhang P, Sun X, Wang F, Kell H: **High-throughput sequencing revealed differences of microbial community structure and diversity between healthy and diseased Caulerpa lentillifera**. *BMC Microbiol* 2019, **19**(1):225.
14. Magoc T, Salzberg SL: **FLASH: fast length adjustment of short reads to improve genome assemblies**. *Bioinformatics* 2011, **27**(21):2957-2963.
15. Bokulich NA, Subramanian S, Faith JJ, Gevers D, Gordon JI, Knight R, Mills DA, Caporaso JG: **Quality-filtering vastly improves diversity estimates from Illumina amplicon sequencing**.

Nat Methods 2013, **10**(1):57-59.

16. Edgar RC, Haas BJ, Clemente JC, Quince C, Knight R: **UCHIME improves sensitivity and speed of chimera detection**. *Bioinformatics* 2011, **27**(16):2194-2200.

17. Wang Y, Guo H, Gao X, Wang J: **The Intratumor Microbiota Signatures Associate With Subtype, Tumor Stage, and Survival Status of Esophageal Carcinoma**. *Front Oncol* 2021, **11**:754788.

18. Quast C, Pruesse E, Yilmaz P, Gerken J, Schweer T, Yarza P, Peplies J, Glockner FO: **The SILVA ribosomal RNA gene database project: improved data processing and web-based tools**. *Nucleic Acids Res* 2013, **41**(Database issue):D590-596.

19. Herr JR, Opik M, Hibbett DS: **Towards the unification of sequence-based classification and sequence-based identification of host-associated microorganisms**. *New Phytol* 2015, **205**(1):27-31.

20. Xu C, Pan D, Zhang D, Lin L, Chen Y, Liang S, He J: **Investigation of the fermentation filtrate from soapberry (Sapindus mukorossi Gaertn.) pericarp on improving the microbial diversity and composition of the human scalp**. *Front Microbiol* 2024, **15**:1443767.

21. Sang SH, Akowuah GA, Liew KB, Lee SK, Keng JW, Lee SK, Yon JA, Tan CS, Chew YL: **Natural alternatives from your garden for hair care: Revisiting the benefits of tropical herbs**. *Heliyon* 2023, **9**(11):e21876.

22. Chiu CH, Huang SH, Wang HM: **A Review: Hair Health, Concerns of Shampoo Ingredients and Scalp Nourishing Treatments**. *Curr Pharm Biotechnol* 2015, **16**(12):1045-1052.

23. Saxena R, Mittal P, Clavaud C, Dhakan DB, Roy N, Breton L, Misra N, Sharma VK: **Longitudinal study of the scalp microbiome suggests coconut oil to enrich healthy scalp commensals**. *Sci Rep* 2021, **11**(1):7220.

24. Saxena R, Mittal P, Clavaud C, Dhakan DB, Hegde P, Veeranagaiah MM, Saha S, Souverain L, Roy N, Breton L *et al*: **Comparison of Healthy and Dandruff Scalp Microbiome Reveals the Role of Commensals in Scalp Health**. *Front Cell Infect Microbiol* 2018, **8**:346.

25. Grimshaw SG, Smith AM, Arnold DS, Xu E, Hoptroff M, Murphy B: **The diversity and abundance of fungi and bacteria on the healthy and dandruff affected human scalp**. *PLoS One* 2019, **14**(12):e0225796.

26. Fang P, Peng F, Gao X, Xiao P, Yang JJo: **Decoupling the Dynamics of Bacterial Taxonomy and Antibiotic Resistance Function in a Subtropical Urban Reservoir as Revealed by High-Frequency Sampling**. 2019, **10**.

27. Li Y, He X, Yuan H, Lv G: **Differed Growth Stage Dynamics of Root-Associated Bacterial and Fungal Community Structure Associated with Halophytic Plant Lycium ruthenicum**. 2022, **10**(8):1644.

28. Dharumadurai Dhanasekaran YJ: **Actinobacteria - Basics and Biotechnological Applications**. London, United Kingdom: InTech Press; 2016.

29. Grime JPJJoE: **Benefits of plant diversity to ecosystems: immediate, filter and founder effects**. 1998.

30. Campetella G, Chelli S, Simonetti E, Damiani C, Bartha S, Wellstein C, Giorgini D, Puletti N, Mucina L, Cervellini M *et al*: **Plant functional traits are correlated with species persistence in the herb layer of old-growth beech forests**. *Sci Rep* 2020, **10**(1):19253.

31. Tsai WH, Fang YT, Huang TY, Chiang YJ, Lin CG, Chang WW: **Heat-killed Lacticaseibacillus paracasei GMNL-653 ameliorates human scalp health by regulating scalp microbiome**. *BMC*

Microbiol 2023, **23**(1):121.

32. Yuanyuan Hao JVSA, K. W. Thilini Chethana, Ishara S. Manawasinghe, Xinghong Li, Mei Liu, Kevin D. Hyde, Alan J. L. Phillips & Wei Zhang: **Nigrospora Species Associated with Various Hosts from Shandong Peninsula, China**. *Mycobiology* 2020, **48(3)**:169-183.
33. Wang M, Liu F, Crous PW, Cai L: **Phylogenetic reassessment of Nigrospora: Ubiquitous endophytes, plant and human pathogens**. *Persoonia* 2017, **39**:118-142.
34. Mc Ginnis MR: **Laboratory Handbook of Medical Mycology**. New York: Academic Press INC; 1980.
35. Manoharachary Cea: **Mycology and microbiology**. India: Scientific Publishers; 2016.
36. Meredith DS: **Atmospheric content of Nigrospora spores in Jamaican banana plantations**. *J Gen Microbiol* 1961, **26**:343-349.
37. Ananya TS, Kindo AJ, Subramanian A, Suresh KJIJoCR, Images: **"Nigrospora Sphaerica" Causing Corneal Ulcer in an Immunocompetent Woman: A Case Report**. 2014, **5**(10):675-679.
38. Motswaledi HM, Pillay RT: **An unusual deep fungal infection with Nigrospora sphaerica in HIV positive patient**. *Int J Dermatol* 2019, **58**(3):333-335.
39. Ma L, Niu Y, Yuan C, Bai T, Yang S, Wang M, Li Y, Shao L: **The Characteristics of the Skin Physiological Parameters and Facial Microbiome of "Ideal Skin" in Shanghai Women**. *Clin Cosmet Investig Dermatol* 2023, **16**:325-337.
40. Chen CH, Liou ML, Lee CY, Chang MC, Kuo HY, Chang TH: **Diversity of nasal microbiota and its interaction with surface microbiota among residents in healthcare institutes**. *Sci Rep* 2019, **9**(1):6175.
41. Peng LH, Liang X, Guo XP, Yoshida A, Osatomi K, Yang JLJMG: **Complete genome of Pseudoalteromonas marina ECSMB14103, a mussel settlement-inducing bacterium isolated from the East China Sea**. 2018:S1874778718300382.
42. Wang JS, Peng LH, Guo XP, Yoshida A, Osatomi K, Li YF, Yang JL, Liang XJMG: **Complete genome of Pseudoalteromonas atlantica ECSMB14104, a Gammaproteobacterium inducing mussel settlement**. 2019.
43. Garlet A, Andre-Frei V, Del Bene N, Cameron HJ, Samuga A, Rawat V, Ternes P, Leoty-Okombi S: **Facial Skin Microbiome Composition and Functional Shift with Aging**. *Microorganisms* 2024, **12**(5).
44. De Ligne L, Vidal-Diez de Ulzurrun G, Baetens JM, Van den Bulcke J, Van Acker J, De Baets B: **Analysis of spatio-temporal fungal growth dynamics under different environmental conditions**. *IMA Fungus* 2019, **10**:7.
45. Shah RR, Larrondo J, Dawson T, Mcmichael A: **Scalp microbiome: a guide to better understanding scalp diseases and treatments**. *Archives of Dermatological Research* 2024, **316**(8):1-9.
46. Cornwell PA: **A review of shampoo surfactant technology: consumer benefits, raw materials and recent developments**. *International journal of cosmetic science* 2018, **40**(1):16-30.
47. Bailey KL, Tilton F, Jansik DP, Ergas SJ, Wellman DM: **Growth inhibition and stimulation of Shewanella oneidensis MR-1 by surfactants and calcium polysulfide**. *Ecotoxicology and Environmental Safety* 2012, **80**(2):195-202.
48. Karabay O, Sahin I: **In vitro activity of sodium-benzoate against isolates of methicillin-resistant Staphylococcus aureus**. *West Indian Medical Journal* 2005, **54**(2):107.

49. Stergaard E: **Evaluation of the antimicrobial effects of sodium benzoate and dichlorobenzyl alcohol against dental plaque microorganisms. An in vitro study**. *Acta Odontologica Scandinavica* 1994, **52**(6):335-345.
50. Dréno B, Zuberbier T, Gelmetti C, Gontijo G, Marinovich M: **Safety review of phenoxyethanol when used as a preservative in cosmetics**. *Journal of the European Academy of Dermatology and Venereology* 2019, **33**(S7):15-24.
51. Wang, Xuewei, Meyerhoff, Mark, E., Ferguson, Stephen, A.: **Detecting levels of polyquaternium-10 (PQ-10) via potentiometric titration with dextran sulphate and monitoring the equivalence point with a polymeric membrane-based polyion sensor**. *Analytical Methods* 2016.
